# Reversible Vitamin B12 Deficiency Presenting with Acute Dementia, Paraparesis, and Normal Hemoglobin

**DOI:** 10.1155/2016/4301769

**Published:** 2016-12-13

**Authors:** Hani Almoallim, Fahtima S. Mehdawi, Mohammed M. Cheikh, Fahmi Al-dhaheri, Abdullah Mahir Aqeel

**Affiliations:** ^1^Alzaidi Chair of Research, Department of Medicine, Umm Al-Qura University, Makkah, Saudi Arabia; ^2^Soliman Fakeeh Hospital, Jeddah, Saudi Arabia; ^3^Department of Medicine, Soliman Fakeeh Hospital, Jeddah, Saudi Arabia; ^4^Umm Al-Qura University, Makkah, Saudi Arabia

## Abstract

Vitamin B12 is essential for neurological function and its deficiency is associated with many neuropsychiatric disorders. We report the case of a previously healthy 53-year-old male patient presenting with delirium and multiple neurological findings. Complete blood analysis indicated megaloblastic anemia. All infectious causes were excluded owing to negative cultures (blood and urine). Tests for human immunodeficiency virus, syphilis, and toxoplasma were also negative. Metabolic workup showed severe vitamin B12 deficiency, decreased reticulocyte count, and increased direct bilirubin and lactate dehydrogenase. Intramuscular injection of cobalamin was started, and the patient showed significant improvement.

## 1. Introduction

Vitamin B12 deficiency is common in developing countries. It can result in neuropsychiatric manifestations such as peripheral neuropathy, myeloneuropathy, cerebellar ataxia, optic atrophy, delirium, dementia, psychosis, or mood disorders [1–3]. Reports of patients initially presenting to psychiatric facilities without associated hematological manifestations are rare. Here, we report a case of vitamin B12 deficiency presenting with a two-month history of delirium symptoms without anemia. The patient responded dramatically to parenteral cobalamin supplementation.

## 2. Case Report

A 53-year-old male patient, not known to have any medical illness, was brought to the emergency room by his wife and daughter due to decreased oral intake since three days. The decreased oral intake was associated with vomiting. The patient had a normal state of health until 2 months prior to his visit, when he started to develop changes in his behavior. According to his wife, the patient became more aggressive, had reduced sleep, had stopped going to work, and became isolated. He also had hallucinations and episodes of short-term memory loss. The above symptoms were accompanied by generalized body weakness, mainly in the lower limbs. The body weakness was associated with pain; the patient became wheelchair-bound. During the last two months, the patient visited many physicians, but no definite diagnosis was determined. He was examined by a psychiatrist and was diagnosed with organic mood disorder. He was prescribed quetiapine (antipsychotic), mirtazapine (antidepressant), and lamotrigine (mood stabilizer). However, according to his wife, he showed no improvements after taking the medications.

Three months prior to his visit, the patient was admitted by the gastroenterology team due to biliary obstruction and endoscopic retrograde cholangiopancreatography (ERCP) was performed; biliary stricture was observed and he underwent stent insertion. At this time, he was also found to have a focal hepatic lesion. He did not have any past psychiatric history, a history suggestive of diabetes mellitus, autoimmune diseases, or any past gastric or ileal surgeries. He had a brief history of alcohol consumption but had stopped drinking 20 years ago. He was not a vegetarian. His daily diet was predominantly carbohydrate-rich and contained adequate animal protein.

An initial examination indicated pale mucous membranes, but no jaundice. The patient was conscious, but disoriented to place, time, and person. He said irrelevant words, was unable to comprehend others, and had bouts of crying. During the neurological examination, he was unable to support himself while standing. All cranial nerves were intact. Fine resting tremor was observed in both hands. The upper limbs had normal muscle size, a power rating of 5/5, normal reflexes, and hypertonia. The upper limb sensory level could not be assessed. The lower limbs had a power rating of 3-4/5 symmetrical, increased reflexes and hypertonia. There was pain in the lower limbs on passive movement and a positive Babinski sign. Senses of vibration and proprioception could not be assessed. Cerebellar examination indicated an abnormal finger-to-nose test and an abnormal shin-to-heel test with dysdiadochokinesia. The patient had a score of 8/30 on the Mini Mental State Examination. The rest of the examination was unremarkable.

The patient's initial laboratory investigations indicated a normal white blood cell count, mildly decreased hemoglobin (12.4 g/dL), elevated mean corpuscular volume (MCV) (102 fL), and normal platelet count (212 × 10^3^ U/L). There were undetectable levels of vitamin B12 (less than 83 pg/mL). His aspartate aminotransferase (AST) and alanine aminotransferase (ALT) levels were mildly elevated at 36 U/L and 44 U/L, respectively. The patient had a lactate dehydrogenase (LDH) level of 264 U/L, total bilirubin of 1.6 mg/dL, and direct bilirubin of 0.48 mg/dL. The serum iron level was 6 *μ*mol/L, and the reticulocyte count was 0.2%. The levels of creatinine, serum electrolytes, and ceruloplasmin and the thyroid function test were normal.  Table 1 shows the results of all lab investigations at the time of presentation of the patient. Tests for human immunodeficiency virus and syphilis and drug screening tests were negative. Magnetic resonance imaging (MRI) of the whole spine was performed and showed osteophytic lesions at the C3-C4, C5-C6, and C6-C7 levels. The spine MRI was normal otherwise. Brain MRI with contrast showed diffuse involutional brain changes and chronic white matter ischemia. An upper endoscopy was performed and was normal. In addition, biopsies showed no evidence of atrophy or malignancy.

During the patient's admission, the neurology team was consulted, and they initially advised to stop all medication. They also requested brain computed tomography (CT) and whole spine MRI. Later, when the diagnosis of vitamin B12 deficiency was established, the neurology team advised that there is no need for further neurological investigations. An abdomen and pelvis CT with contrast was performed for further evaluation of the focal hepatic lesion and showed multiple focal hepatic cystic lesions with normal liver enzymes. The patient is being followed up by the gastroenterology team.

The patient was started on 1 mg intravenous methycobalamin (1,000 mcg) every day for two weeks. The dosage was then changed to 1 mg once weekly by intramuscular injection for another month after discharge. The patient was then switched to 500 mcg oral cobalamin three times daily for life. He returned for follow-up three months later.

Two months after starting the treatment, the patient came back for a follow-up. There was a major change in his behavior. He was fully oriented and had significant improvements in memory, but he did not remember when he was admitted. Power in lower limbs was 3/5 and he had an unsteady gait. Five months after starting the treatment, the patient returned for a second follow-up and had marked improvements in gait, muscle power, and cognitive function. Six months after starting the treatment, the patient was able to walk alone, drive his car, and had lower limb power of 5/5. The Mini Mental score was reassessed and was 30/30. The patient provided informed consent for his case to be reported.

## 3. Discussion

We present the case of a middle-aged man, with no previous history of psychiatric illness, who presented with acute dementia and paraparesis since two months with normal hemoglobin levels. He visited many physicians during this time and was diagnosed with organic mood disorder. The patient was prescribed antipsychotic medication but showed no improvement. The patient's acute presentation of delirium and profound neuropsychosis was the main obstacle for early diagnosis. Further diagnostic workup indicated vitamin B12 deficiency with evidence of ineffective erythropoiesis. The patient had a remarkable response to vitamin B12 replacement, and all antipsychotic medications were stopped.

Vitamin B12 deficiency is usually the result of inadequate absorption, as in the cases of pernicious anemia, gastric disease, or inadequate intake [4–7]. It is widely known for its hematological and neurological manifestations but usually does not present with predominantly psychiatric manifestations, as in this case. Neuropsychiatric manifestations of vitamin B12 deficiency include dementia, delirium, cerebellar ataxia, psychosis, neuropathy, and mood disorders. Acute delirium is rarely the only presenting symptom. In our case, acute delirium preceded the typical hematological and neurological findings observed in vitamin B12 deficiency. Patients presenting with neuropsychiatric symptoms due to vitamin B12 deficiency have a less severe presentation of megaloblastic anemia [8]. Severe megaloblastic anemia is seldom accompanied by any neurological symptoms or signs. The underlying reason for this inverse relationship remains unclear. Vitamin B12 acts as a coenzyme for L-methylmalonyl-coenzyme A mutase and methionine synthetase. Thus, enzymatic defects resulting from vitamin B12 deficiency lead to accumulation of methylmalonic acid and homocysteine, which appear to be proportionally related to the severity of the associated neurological and psychiatric abnormalities [9].

There are multiple factors that lead to misdiagnosis of vitamin B12 deficiency. Most physicians are not aware that psychiatric symptoms may sometimes be the only presenting symptoms of vitamin B12 deficiency. Most doctors depend on MCV and MCH values to diagnose vitamin B12 deficiency. However, perturbations in MCV and MCH are late signs of vitamin B12 deficiency. In addition, the serum vitamin B12 test is not very sensitive or specific. At the same time, serum vitamin B12 levels need not to be very low in order to produce psychiatric symptoms [10]. It has been shown that vitamin B12 levels become deficient in neuronal tissue before deficiency is evident in the serum [11]. In such cases, homocysteine and methylmalonic acid assays increase the specificity of the diagnosis of B12 deficiency [12]. However, since these lab tests are not available in our hospital, we confirmed our diagnosis using the treatment response. The patient had dramatic clinical improvement and MCV dropped to 79 fL.

It is vital for physicians to approach each patient with a sense of clinical reasoning [13]. There has been an evolving discussion in the literature of the dual process theory of clinical reasoning. The dual process theory consists of two components: intuition and analytical thinking [14]. This case justifies the use of intuition and “fast thinking.” It not only led us to think outside of the box, but to also reconsider the patient's initial diagnosis. On the other hand, the use of analytical thinking or “slow thinking” allowed us to gather information regarding all of the features of the patient and to determine a differential diagnosis. Our patient was examined by many physicians from many specialties who were all focused on one diagnosis without considering alternatives despite the lack of response to treatment. It is easy to label a patient with a specific diagnosis, but the challenge is to justify this diagnosis and to confirm it. This may be achieved by following a rigorous model in clinical reasoning.

Although vitamin B12 deficiency is common, it may sometimes be overlooked. The most common known manifestation of vitamin B12 deficiency is megaloblastic anemia. The purpose of this case report is to show that if vitamin B12 deficiency remains undiagnosed, serious sequelae may occur. Assessment of B12 levels should be included as a standard evaluation in patients presenting with new onset of depressive disorders, acute delirium, dementia, or psychosis. Therefore, prevention, early detection, and management of this reversible state are of profound importance.

## Figures and Tables

**Table 1:** Lab investigations at time of presentation.

Lab investigation	Result	Normal values
WBC	9.2 × 10^3^/*μ*L	(4.3–10.3)10^3^/*μ*L

Hemoglobin	12.4 g/dL	(13–18) g/dL
MCV	102 fL	(80–96) fL
Mean corpuscular hemoglobin	34 pg	(27–34) pg

Platelet	212 ×10^3^/*μ*L	(150–400)10^3^/*μ*L

Sodium	144 mmol/L	(136–145) mmol/L

Potassium	5.1 mmol/L	(3.5–5.1) mmol/L

Creatinine	0.89 mg/dL	(0.73–1.18) mg/dL

Total creatinine kinase	1,588 U/L	(30–200) U/L

AST	36 U/L	(5–34) U/L

ALT	44 U/L	(0–55) U/L

Total bilirubin	1.6 mg/dL	(0.2–1.2) mg/dL
Direct bilirubin	0.482 mg/dL	(0–0.5) mg/dL

LDH	264 U/L	(125–220) U/L

Vitamin B12	<83 pg/mL	(187–883) pg/mL

Serum iron	6.9 *μ*mol/L	(11.6–31.3) *μ*mol/L

Reticulocyte count	0.2%	(0.5%–3.5%)
